# Mesenchymal cystic hamartoma of the lung: A case report

**DOI:** 10.1097/MD.0000000000028242

**Published:** 2022-01-07

**Authors:** Ligong Yuan, Shuaibo Wang, Jiacong Wei, Kun Yang, Yousheng Mao

**Affiliations:** a Department of Thoracic Surgery, National Cancer Center/National Clinical Research Center for Cancer/Cancer Hospital, Chinese Academy of Medical Sciences & Peking Union Medical College, Beijing, China; b Department of Pathology, National Cancer Center/National Clinical Research Center for Cancer/Cancer Hospital, Chinese Academy of Medical Sciences & Peking Union Medical College, Beijing, China.

**Keywords:** lung, mesenchymal cystic hamartoma, misdiagnosis

## Abstract

**Rationale:**

Mesenchymal cystic pulmonary hamartoma is a rare type of hamartoma that has been reported in all cases in the literature. Most patients were reported to have spontaneous pneumothorax and were treated by surgery, and finally confirmed to be caused by rupture of the cystic hamartoma. Here, we report a case of mesenchymal cystic pulmonary hamartoma detected using computed tomography (CT) during a health check-up without obvious symptoms.

**Patient concerns:**

A 60-year-old woman was detected using CT during her health check-up. She was a non-smoker and had no symptoms or history of specific diseases.

**Diagnosis:**

The final pathological examination confirmed that the lesion was a mesenchymal cystic hamartoma of the lung.

**Interventions:**

A uniportal video-assisted thoracic surgery wedge resection was performed for biopsy.

**Outcomes:**

The patient recovered smoothly and was discharged on postoperative day 3.

**Lessons:**

For cystic pulmonary hamartoma, it is usually difficult to make a correct diagnosis using CT imaging. A chest magnetic resonance imaging examination may be helpful for differentiation diagnosis before video-assisted thoracic surgery biopsy.

## 1 Introduction

As the most common benign tumor removed surgically from the lung, hamartoma accounts for approximately 5% to 10% of the single solid nodule or tumor in the lung, secondary only to lung cancer and pulmonary inflammatory granulomas.^[1]^ Most hamartomas grow slowly,^[2]^ and they can be located peripherally (peripheral type) or centrally (central type). The former usually occurs in the peripheral lung field and distal to the segmental bronchus, and the latter frequently occurs within the lung segment and close to the segmental bronchus. Most hamartomas have no obvious clinical symptoms and are occasionally discovered on a health check examination. However, once the lesion compresses the surrounding bronchus, symptoms such as fever, cough, and chest pain may present.^[3]^ Pathologically, pulmonary hamartoma is composed of mature cartilage, mixed with adipose tissue, smooth muscle tissue, mucinous fibrous connective tissue, ciliated epithelium, irregular fissures, and adenoid structures lined by the respiratory epithelium. These components are usually mixed to various extents.^[4]^ Hamartoma originates from the connective tissue of the mesenchymal lobe of the bronchus. According to the dominant histological composition of the originated tissues, hamartomas can be further divided into several subtypes, including chondroma, smooth muscle, and connective tissue. Among these, chondroma hamartoma is the most common.^[5]^ Chondroma hamartomas usually appear as a solid, rounded, and non-lobulated nodule with a clear and smooth edge on CT scan. Its typical radiological features are popcorn-like calcification and fat density shadows in the lesion, while approximately 50% of hamartomas have no calcification or fat density in the lesions. Other types of nodules, including peripheral lung cancer, tuberculoma, metastatic cancer nodules, and inflammatory pseudotumors, are the lesions most frequently observed and need to be differentiated from hamartomas during clinical practice, most of which do not contain fat components.^[6]^

## 2 Case report

**Figure 1:**
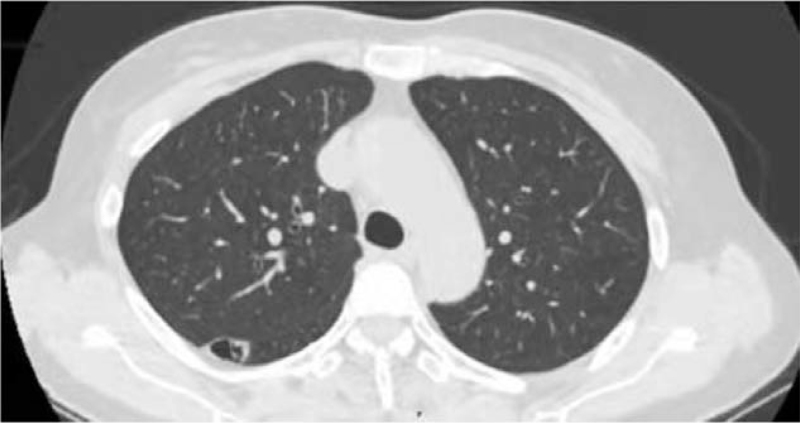
Chest computed tomography scan: the nodule with a thin wall bulla was located in the dorsal segment of the right lower lobe, with a size of 2.0 × 0.8 cm.

**Figure 2:**
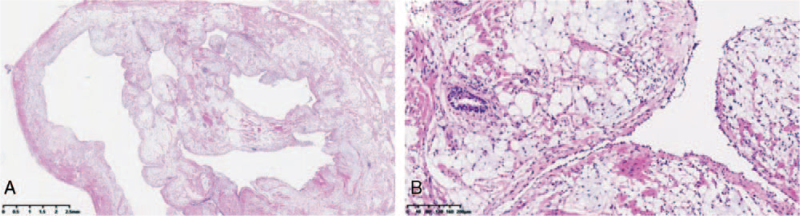
A, Indicates the HE histopathological changes of the hamartoma in the low-power field at 10× magnification, in which a well-circumscribed cystic tumor could be observed abutting the lung tissue. B, Shows a mixture of mature adipocytes and smooth muscle cells with entrapped clefts lined by a single layer of respiratory epithelium and local myxoid changes in a high-power field at 100× magnification.

A single nodule in the lower lobe of the right lung in a 60-year-old woman was detected using computed tomography (CT) during her health check-up. The nodule with a thin wall bulla was located in the dorsal segment of the right lower lobe, with a size of 2.0 × 0.8 cm, (Fig. 1). There were no other obvious abnormalities in the respiratory systems. She was a non-smoker and had no symptoms or history of specific diseases. There were no significant changes in size and features on chest CT after 2 weeks of oral antibiotic treatment to rule out the possibility of pneumonia. Eventually, the patient was transferred to our hospital for diagnosis and treatment. A preoperative pulmonary function test suggested that her pulmonary function was sufficient to undergo surgical resection. There were no obvious abnormalities in the tumor markers. Then, uni-port video-assisted thoracic surgery (VATS) wedge resection through the left fifth intercostal incision was performed for biopsy. During the operation, the nodule was found to be a cystic thin-walled nodule with a size of 1.8 × 0.9 × 0.6 cm, which was filled with light yellow clear liquid. And the size of the gross specimen was 5.5 × 3.0 × 1.5 cm. The intraoperative frozen section revealed a hamartoma. The patient recovered smoothly and was discharged on postoperative day 3. The final pathological examination confirmed that the lesion was a mesenchymal cystic hamartoma of the lung (a mixture of mature adipocytes and smooth muscle cells with entrapped clefts lined by a single layer of respiratory epithelium, Fig. 2A and B). Four months after surgery, a chest CT scan showed no recurrence.

## 3 Discussion

Mesenchymal cystic pulmonary hamartoma is a rare type of hamartoma that has been reported in all cases in the literature. Most patients were reported to have spontaneous pneumothorax and were treated by surgery, and finally confirmed to be caused by rupture of the cystic hamartoma.^[7–9]^ Most mesenchymal cystic hamartomas of the lungs are asymptomatic. Some of these patients have atypical clinical manifestations, such as wheezing, hemoptysis, pneumothorax, and hemothorax. Among these symptoms, recurrent spontaneous pneumothorax is the most commonly documented.^[10]^ Cystic hamartoma is likely to become a cyst when the diameter is >1 cm,^[11]^ and the cyst is lined with ciliated columnar epithelial cells or metaplastic squamous epithelial cells. The mechanism by which lung cystic hamartomas develop into cysts remains unclear. Intra-cystic necrosis due to insufficient blood supply, bleeding, or secreted mucin may cause liquefaction of the cystic hamartoma. The imaging features of cystic hamartomas usually present as cystic pulmonary nodules with irregular shapes, partition formation, and uneven thickening of the cyst wall with partition. The lack of specific image characteristics on CT scans is the main reason for misdiagnosis. Most of these nodules were not necessary for surgical resection with a good prognosis. The nodule presented as a cystic nodule and a thin wall. Therefore, this patient was suspected to have lung cancer, and a VATS biopsy was performed to confirm the diagnosis. During the operation, the nodule was found to be a cystic thin-walled nodule, which was filled with a clear lightly yellow liquid. The final diagnosis was mesenchymal pulmonary hamartoma by pathology, in which smooth muscle and adipose tissue were found, while no mature cartilage structure and calcification were detected, which led to misdiagnosis in the patient due to lack of specific imaging features. For cystic pulmonary hamartoma, it is usually difficult to make a correct diagnosis through CT-guided fine needle biopsy because most of the harvested tissue is the fluid content of the cyst, rather than the tissue of the thin cystic wall. A chest magnetic resonance imaging examination may be helpful for differentiation diagnosis before VATS biopsy.

## Author contributions

**Data curation:** Ligong Yuan, Shuai bo Wang.

**Formal analysis:** Ligong Yuan, Shuai bo Wang.

**Resources:** Shuai bo Wang, Jiacong Wei, Kun Yang.

**Writing – original draft:** Ligong Yuan.

**Writing – review & editing:** Yousheng Mao.
